# Monitoring *Colletotrichum* Colonization and Reproduction in Different Rubber Tree Clones

**DOI:** 10.3390/plants11070905

**Published:** 2022-03-29

**Authors:** Ana Carolina Firmino, Izabela Ponso Magalhães, Marcela Eloi Gomes, Ivan Herman Fischer, Erivaldo José Scaloppi Junior, Edson Luiz Furtado

**Affiliations:** 1 College of Agricultural and Technological Sciences, São Paulo State University (Unesp), Dracena 17900-000, Brazil; izabelaponso@gmail.com (I.P.M.); marcelaeloi14g@gmail.com (M.E.G.); 2 Central-West Regional Center, São Paulo’s Agency for Agribusiness Technology (APTA), Bauru 17030-000, Brazil; ihfische@gmail.com; 3 Center of Rubber Tree and Agroforestry Systems, Agronomic Institute of Campinas (IAC), Votuporanga 15505-970, Brazil; scaloppijr@yahoo.com.br; 4 School of Agriculture, São Paulo State University (Unesp), Botucatu 18610-034, Brazil; edson.furtado@unesp.br

**Keywords:** *Colletotrichum*, *Hevea brasiliensis*, cycle

## Abstract

Anthracnose, caused by fungi of the genus *Colletotrichum*, is present in the major rubber tree crop areas in Brazil, especially in São Paulo, Mato Grosso do Sul, Paraná, Minas Gerais, Espírito Santo, and northern states. This disease can affect different tissues of the rubber tree, leading to production losses. Thus, a better understanding of the pathosystem *Colletotrichum* x rubber tree can provide evidence to subsequent epidemiological research and phytosanitary management studies of this disease in the field. The present study aimed to investigate *C.* *tamarilloi* colonization and reproduction steps in resistant clones (IAC 502, IAC 507, RRIM 937) and in one susceptible clone (RRIM 600) of the rubber tree, verifying the influence of temperature up to 48 h after inoculation of the fungus, under 24 h wetness. Samples were analyzed under a light, a UV and a scanning electron microscope. Data indicated that the fungus had a delay in its development in resistant clones and, although colonization was expressive 48 h after inoculation, the new spore formation rate in the analyzed samples was lower in resistant clones. For RRIM 600, rapid colonization and intensive sporulation could be observed.

## 1. Introduction

The rubber tree is a species grown in different places of Brazil, from Paraná State to the north region [1]. However, there are some limiting factors to increase the latex productivity of Brazilian rubber tree crops, including phytosanitary problems caused by several diseases such as anthracnose. This disease, caused by several species of *Colletotrichum*, is responsible for affecting different tissues of the rubber tree, but new sproutings show symptoms with higher frequency. A severe attack causes defoliation, descending branch drought and apical bud death [2].

Among the causal agents of anthracnose already reported in Brazil are *C. gloeosporioides* and *C. acutatum* [2]. However, new studies indicate that there are other species of this fungus associated with anthracnose in rubber trees, such as the species *C. fructicola* and *C. tamarilloi* [3,4]. Therefore, information about these last two species in rubber trees is limited in the literature.

The species *C. tamarilloi* was described by Damm et al., 2012 [5] from fungal isolates obtained from *Solanum betaceum* fruits with anthracnose from Colombia. In Brazil, this fungus was first reported in *Solanum* gilo plants in 2014 [6] and now in rubber trees in 2021 [6]. This species of *Colletotrichum* belongs to the complex of species *C. acutatum*, and its spores are asexual, conidial, formed in conidiophores coming directly from the hyphae. The formation of salmon-colored mucilage is described in this species, which is important for the protection of spores and acervuli under unfavorable environmental conditions for the fungus. This mucilage also has the function of facilitating the dissemination of spores by drops of water [1,5].

Nowadays, disease control is primarily based on fungicide application in nurseries [1,7]. In the field, this method can be limited by the low uniformity of tree canopies and the epidemic stage, which cannot be advanced [8].

Research addressing rubber tree resistance to anthracnose is progressing [3,9]. There is evidence that the resistance of this plant to anthracnose is of the quantitative type since degrees of disease were reported in the clones tested. However, it should be considered that the environment has a strong influence on the expression of this type of resistance and can make the plant more susceptible to attack. Thus, knowing the optimal environmental characteristics for the development of the fungus species associated with anthracnose in different plant varieties, clones or cultivars is extremely important for the management of the disease in the field [10,11].

In addition, different *Colletotrichum* species are known to alter their cycle depending on the host and on the environmental conditions to which they are exposed [12]. Guyot et al. [13] reported that secondary leaf fall in rubber trees and *Colletotrichum* sporulation are closely related to the wetness duration to which the fungus is exposed, and more severe symptoms could be observed after 14-h humidity. Temperature is another factor that determines the behavior and the development of the pathogen; as shown by King et al. [14], sporulation of different *Colletotrichum* species such as *C. acutatum*, *C. gloeosporioides* and *C. fragariae* in strawberry increases over time and at temperatures superior to 15 °C, peaking within the range between 15 and 30 °C, while the sporulation rate slightly differs among the distinct species of the pathogen.

The development of anthracnose in rubber trees, caused by *C. gloeosporioides* and *C. acutatum*, is favored by relative humidity above 90% during 13 daily hours and mean temperatures between 26 and 32 °C [1,15]. According to Magalhães et al. [16], temperatures between 25 and 35 °C and 24 h wetness were favorable to *C. tamarilloi* germination and appressorium formation in resistant and susceptible clones, but the fungus showed different development degrees.

Therefore, the present study aimed to verify the influence of the environment on *C. tamarilloi* colonization and reproduction in clones presenting reactions of resistance (IAC 507, IAC 502 and RIMM 937) and susceptibility (RRIM 600).

## 2. Materials and Methods

### 2.1. Preparation of Materials from Rubber Trees and C. tamarilloi Isolate

The folioles used in this study were collected from the middle portion of the plants (phenological stage D of the leaf [1]) of the RRIM 600, IAC 507, IAC 502 and RRIM 937 clones. These clones present reactions of resistance (IAC 507, IAC 502 and RRIM 937) and susceptibility (RRIM 600), according to Antonio et al. [3]. The plants used in the experiments are located at the clonal garden of Agronomic Institute-IAC, in Votuporanga, São Paulo State, Brazil; they are 7 years old, on average, and spaced at 1.5 m between lines and 0.7 m between plants.

The *C. tamarilloi,* isolate CH09, used in this study was molecularly characterized through sequencing of part of ITS-5.8S rDNA region (MW031267), part of β-Tubulin gene region (OK258095), part of glyceraldehyde-3-phosphate dehydrogenase (GAPDH) gene (OK258094) and part of calmodulin (CAL) gene (OK258093) [17,18,19], and was preserved at the forest pathology fungal collection of FCAT/UNESP. The inoculum used in the experiments was obtained from 7-day culture of the isolates in an oat medium, at 25 ± 1 °C and continuous photoperiod. The conidial suspension in distilled water was adjusted to the concentration of 10^5^ conidia/mL with a Neubauer chamber.

Leaflets of different rubber tree clones were collected from the apical part of the plants, superficially disinfected with NaClO 2% and washed with sterile water. Then, inoculation was carried out by deposition of 30 µL of the conidia suspension (10^5^ conidia/mL) of the isolate on a leaf area of 1 cm^2^ (1 × 1 cm), delimited with plastic adhesives.

After inoculation, the leaflets were incubated in a humid chamber for 24 h, at 15, 20, 25, 30, 35 and 40 ± 1 °C, in the dark. Circular samples of 5 mm in diameter were taken from the inoculated areas at predetermined intervals of the 12, 24, 36 and 48 h (h).

### 2.2. Preparation and Evaluation of Samples under a Light Optical Microscope

Fungal colonization and reproduction in the internal part of the leaf were observed in this evaluation for all tested periods and temperatures. At the end of each predetermined time interval, the collected circular samples were processed according to the methodology described by Celio and Hausbeck [20]. For fixation, leaf discs were placed on filter paper saturated with formalin/alcohol/acetic acid solution (1:18:1 *v*/*v*/*v*) in a plastic Petri plate sealed with Parafilm for 2 h. Then, the discs were clarified about 48 h in a solution containing chloral hydrate (200 g), dH_2_O (80 mL), ethanol (250 mL) and four drops of Tween-20. Subsequently, they were placed in flasks containing 4 mL lactophenol solution (20 g phenol, 20 mL lactic acid, 40 g glycerin and 20 mL water) and lactophenol cotton solution (100 mL lactophenol, 1 mL 1% aqueous solution of cotton blue and 20 mL glacial acetic acid) and were mounted in glycerol for observation under a light microscope.

### 2.3. Preparation and Evaluation of Samples under Ultraviolet Light Microscopy

To complement the study, fungal colonization and reproduction on the leaf were observed through the UV light technique. At the end of each predetermined time interval, circular samples from leaves that were exposed to 25 °C (this temperature was chosen for providing the best fungal development) were collected and processed according to the methodology described by Celio and Hausbeck [20], modified. The sample processing steps were the same as those described in the previous item, except for staining in lactophenol cotton blue solution, which was substituted for Calcofluor White (Sigma^®^, Sigma; Kenilworth, NJ, USA). This reagent (approximately 50 µL) was placed onto the samples which, after one minute, were washed in distilled water (twice for 30 s) and mounted in distilled water.

### 2.4. Preparation and Evaluation of Samples under a Scanning Electron Microscope (SEM)

The disinvolvement of the fungi on the rubber tree leaf different clones was evaluated from leaf fragments of approximately 5 mm in diameter, collected from each clone and fixed in “Karnovsky” (2.5% glutaraldehyde, 2.0% paraformaldehyde and 0.05 M buffer phosphate pH 7.2) for a minimum period of 24 h. Then, the samples were removed from the fixative, fragmented and transferred to 1.5 mL microtubes containing sodium phosphate buffer 0.05 M for ten minutes. Subsequently, the samples were dehydrated in a series of increasing acetone concentrations (30, 50, 70, 90 and 100%) for 10 min each and dried in a critical point device (Bal-Tec CPD 030). After drying the samples, the samples were processed according to the methodology described by Firmino et al. [21]. They were mounted on stubs with double-sided carbon tape and covered with 20 nm gold in a Bal-Tec SCD 050 sputter coater [19], for analysis under a LEO435-VP scanning electron microscope, located at the Center for Electron Microscopy of Luiz de Queiroz College of Agriculture (ESALQ), University of São Paulo (USP), Piracicaba, São Paulo State, Brazil.

### 2.5. Data Analysis

The experiments were carried out in a completely randomized design with 10 replications per treatment, with each treatment consisting of a clone at an evaluation time at one temperature. A photo of each repetition of each treatment was captured to quantify the fungal structures.

The quantitative evaluation of fungal colonization and reproduction, based on the structures produced by the fungus (mycelium and spores) in the leaf, was performed with the software Basic Intensity Quantification with ImageJ [22] with the images captured under ultraviolet light microscopy. The data obtained were submitted to Scott–Knott test at a 5% probability level using the software Sisvar [23].

For the images captured under a light microscope and SEM, a descriptive methodology was adopted, based on the observation of the fungus development in the different clones after inoculation [4,24].

## 3. Results and Discussion

**Figure 1:**
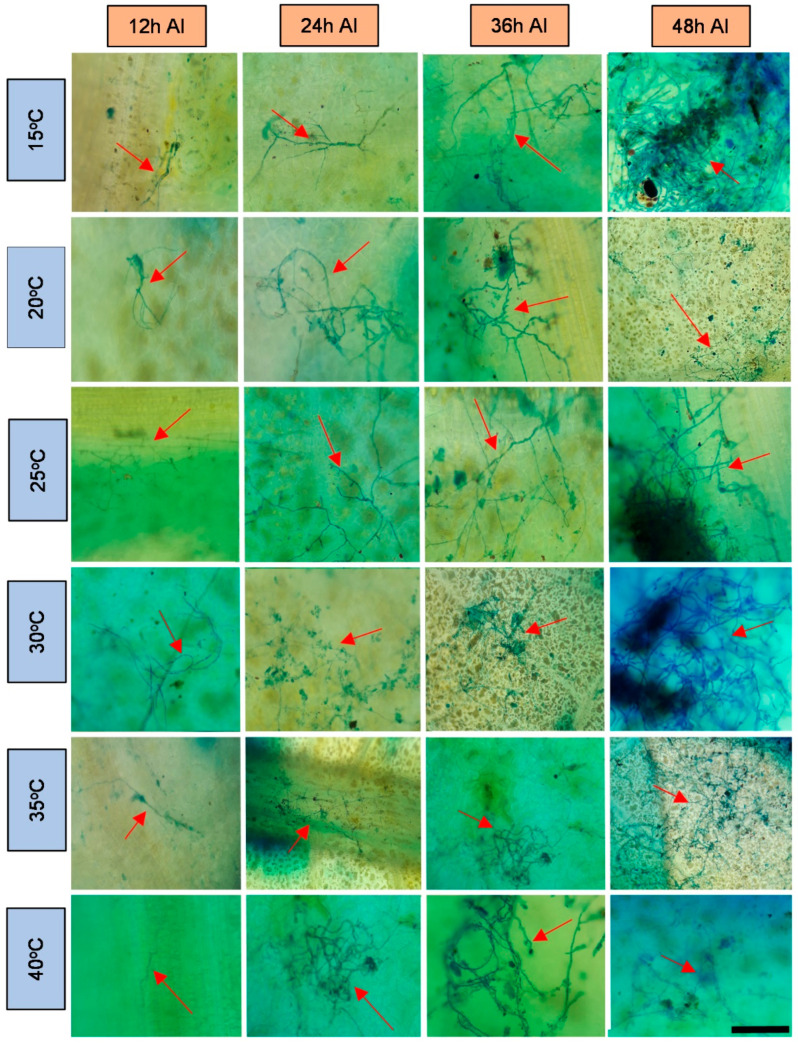
Light microscopy photos of the rubber tree clone RRIM 600 after 24-h wetness, at different temperatures (°C), 12, 24, 36 and 48 h after inoculation (AI) of *C. tamarilloi* stained with lactophenol cotton blue solution (blue color in the photo). The arrow indicates the fungal structures in the plant (mycelium and spores). Bar = 100 µm for all pictures.

**Figure 2:**
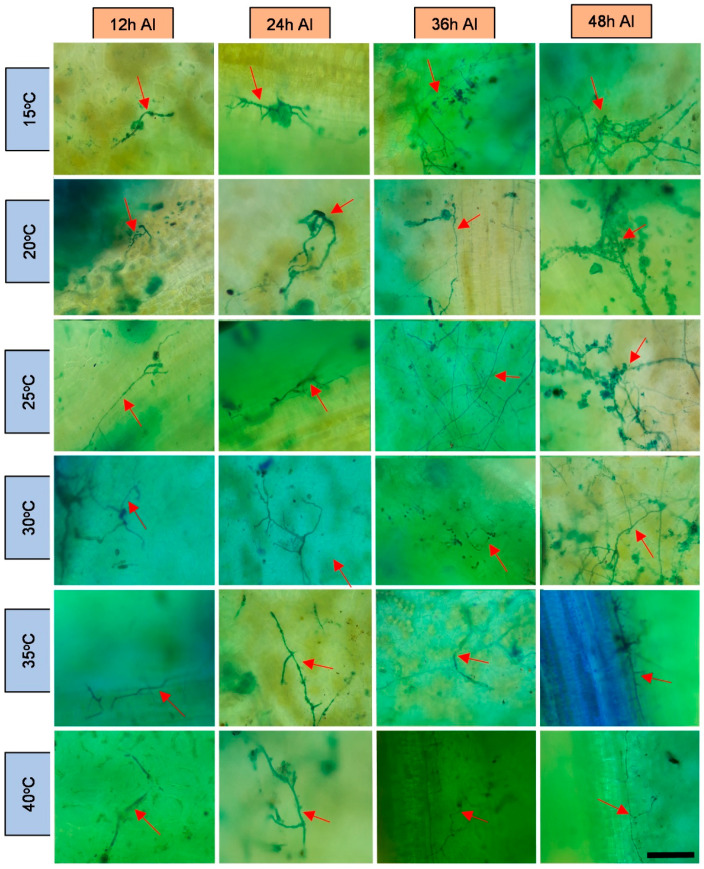
Light microscopy photos of the rubber tree clone RRIM 937 after 24-h wetness, at different temperatures (°C), 12, 24, 36 and 48 h after inoculation (AI) of *C. tamarilloi* stained with lactophenol cotton blue solution (blue color in the photo). The arrow indicates the fungal structures in the plant (mycelium and spores). Bar = 100 µm for all pictures.

**Figure 3:**
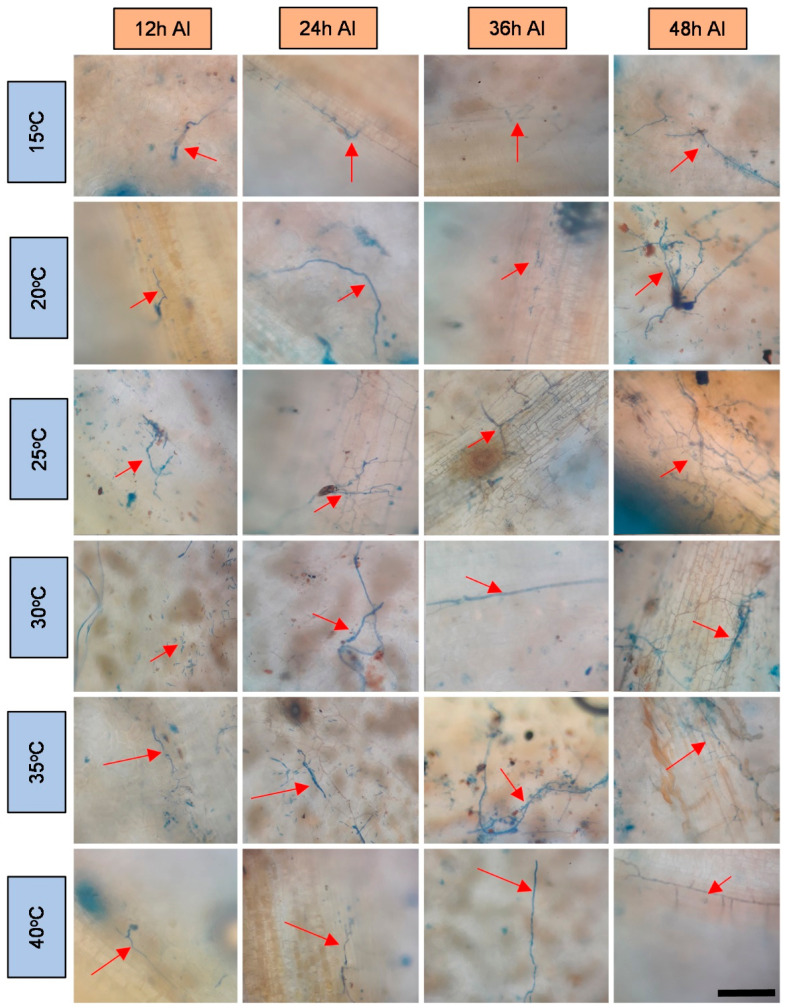
Light microscopy photos of the rubber tree clone IAC 507 after 24-h wetness, at different temperatures (°C), 12, 24, 36 and 48 h after inoculation (AI) of *C. tamarilloi* stained with lactophenol cotton blue solution (blue color in the photo). The arrow indicates the fungal structures in the plant (mycelium and spores). Bar = 100 µm for all pictures.

**Figure 4:**
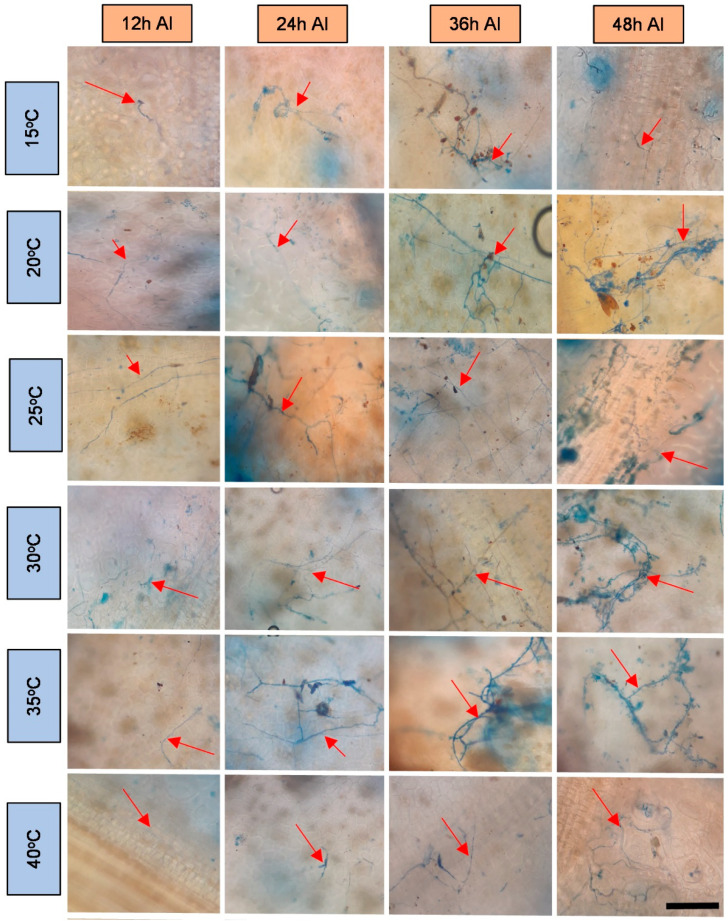
Light microscopy photos of the rubber tree clone IAC 502 after 24-h wetness, at different temperatures (°C), 12, 24, 36 and 48 h after inoculation (AI) of *C. tamarilloi* stained with lactophenol cotton blue solution (blue color in the photo). The arrow indicates the fungal structures in the plant (mycelium and spores). Bar = 100 µm for all pictures.

**Figure 5:**
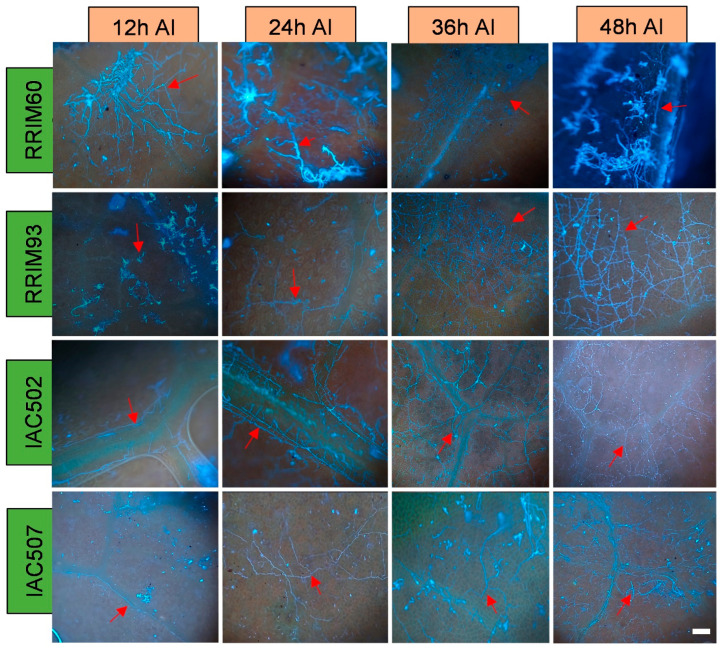
Ultraviolet light microscopy (UV) photos of the rubber tree clones RRIM 600, RRIM 937, IAC 502 and IAC 507, after 24 h wetness at 25 °C, and 12, 24, 36 and 48 h after inoculation (AI) of *C. tamarilloi* (blue fluorescence in the photo). The arrow indicates the fungal structures in the plant (mycelium and spores). Bar = 100 µm for all pictures.

**Figure 6:**
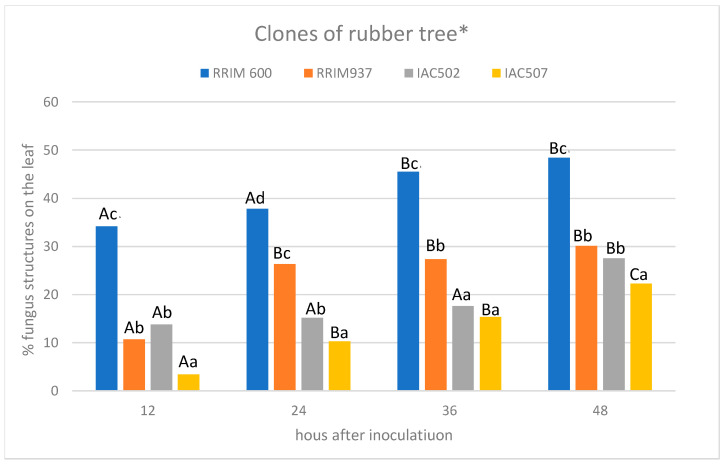
Average percentage of rubber tree leaf area with structures (mycelium and spores) of *C. tamarilloi* at different times after inoculation. * Mean values followed by the same capital letter did not differ in the same clone at different times after inoculation. Mean values followed by the same lowercase letter did not differ between clones at the same time after inoculation. (Scott–Knott test; *p* < 0.05, coefficient of variation: 46.3).

**Figure 7:**
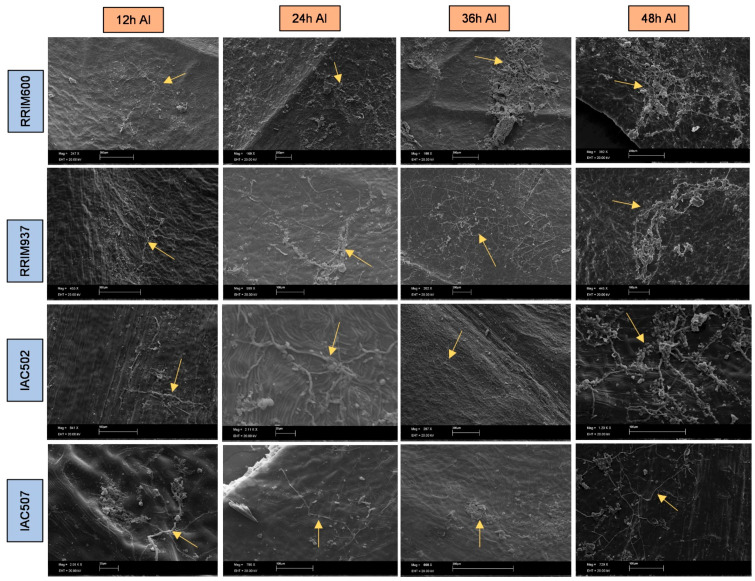
SEM photos of the rubber tree clones RRIM 600, RRIM 937, IAC 502 and IAC 507 after 24 h wetness, at 30 °C, 12, 24, 36 and 48 h after inoculation (AI) of *C. tamarilloi.* The arrow indicates the fungal structures in the plant (mycelium and spores).

The obtained microscope images (Figure 1, Figure 2, Figure 3, Figure 4, Figure 5, Figure 6 and Figure 7) corroborate the findings of Magalhães et al. [4] and reinforce the possibility that the latent period of the fungus is shorter in the susceptible clone than in resistant clones. Magalhães et al. [16] reported that, although all clones showed hyphae on their leaf surfaces after 24-h wetness, RRIM 600 had more advanced development and the *C. tamarilloi* appressorium formation percentage was inferior in resistant clones.

The present paper is evidence that, besides the germination and appressorium formation studied by the above-mentioned authors, colonization and reproduction were more advanced in the susceptible clone (RRIM 600) from 12 h after inoculation, compared to the other clones that have a resistance degree, especially considering UV and SEM images (Figure 5 and Figure 7). These two techniques allowed clear visualization of new spore formation 24 h after inoculation of the fungus in the leaf, highlighting its more expressive reproduction in the clone RRIM600.

**Figure 8:**
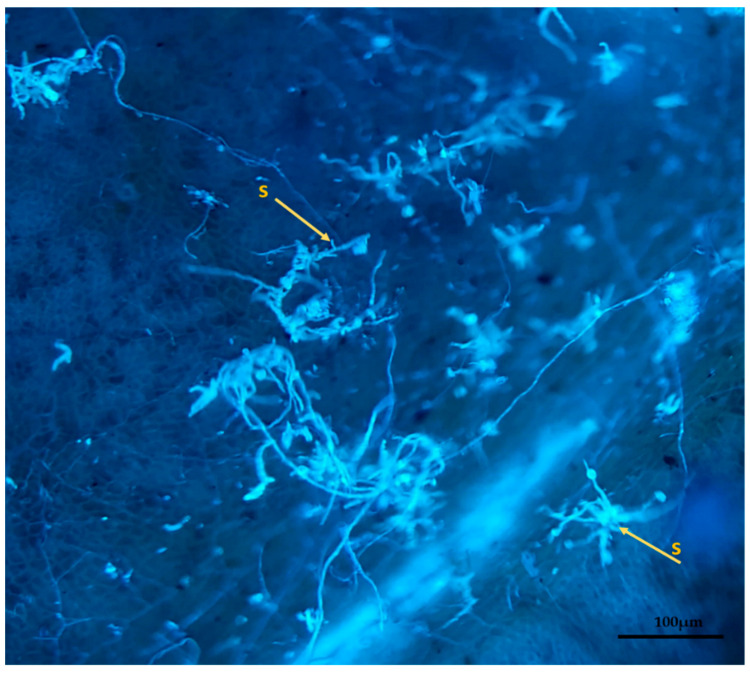
Clone RRIM 600, showing conidiophore of *C. tamarillo* with conidiogenic cells and new spores (s) in ultraviolet light microscopy.

**Figure 9:**
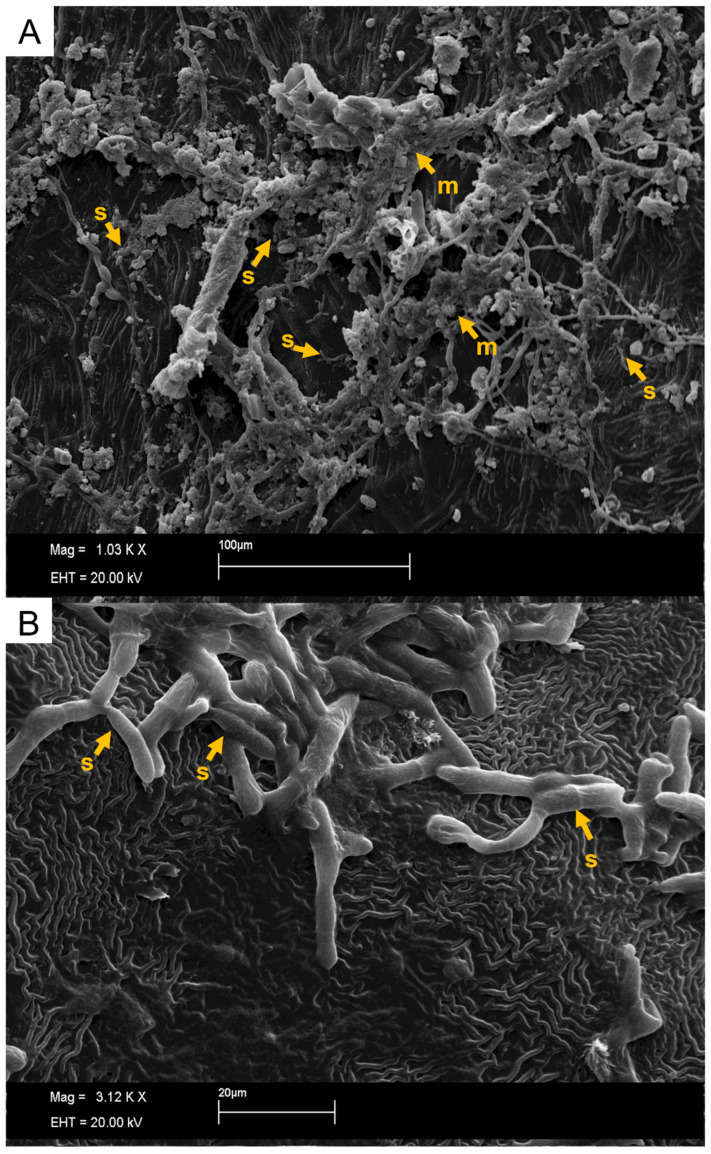
(**A**) Clone RRIM 600, showing conidiophore of *C. tamarillo* with conidiogenic cells with new spores (s) and with mucilage start formation (m) in SEM. (**B**) More details of the *C. tamarillo* with conidiogenic cells with new spores (s).

The SEM and ultraviolet images evidenced that not only new spores were produced in conidiophores coming directly from the hyphae, but also a mass was formed around spores as the time after inoculation increased (Figure 8 and Figure 9), especially for the most susceptible clone after 36 h of the inoculation. This mass can be mucilage which is normally produced by this genus of fungus with the aim of protecting spores for dissemination through raindrops [25,26]. These same characteristics of mucilage formation were described by XIE et al. [27]. The developmental delay in *C. tamarillo* colonization and reproduction in the resistant clones tested here (IAC 507 and IAC 502 and RRIM 937) can be associated with the greater accumulation of lignins, lipids and arginines, and especially with the activity of peroxidase. This enzyme catalyzes the oxidation and polymerization of alcohol hydroxycinnamic acid in the presence of hydrogen peroxide, resulting in lignin production and deposition, as well as participating in the oxidation of phenolic compounds, which accumulate in response to infection [28,29]. Protein level and stomatal density were also lower in resistant clones (IAC 507 and IAC 502 and RRIM 937), compared to RRIM600, which is susceptible [16].

As detected for the pathosystem studied here, *C. tamarilloi*-rubber tree, such a delay in fungal development in resistant plant materials has also been reported by other studies involving *Colletotrichum*. The pathosystem *C. sublineola*-sorghum (*Sorghum bicolor*) has been extensively investigated and can be cited as an example. As found by Magalhães et al. [16] for rubber trees, the initial defense response to anthracnose seems to be similar between resistant and susceptible genotypes in sorghum [30].

Based on the histological studies of the pathosystem sorghum-*C. sublineolum*, the rapid colonization and reproduction of *Colletotrichum* in susceptible rubber trees may be due to the survival of epidermal cells soon after the initial penetration, forming a biotrophic interaction, which is one of the nutrition phases of this fungus. In resistant plants, pigmented cytoplasm rapidly accumulates with inclusions containing phytoalexins that lead to the death of infected cells and the rupture of fungal cytoplasm, preventing thus the development of hyphae [31]. In resistant sorghum plants, primary hypha proliferation in neighboring cells was only observed after the establishment of a biotrophic interaction in the first colonized cells. This initial biotrophic phase can allow the fungus to establish in the tissue to sufficiently decrease the inhibitory effects of the defense compounds, distinguishing between resistant and susceptible plants for germination, penetration, colonization, and reproduction processes of the fungus. However, cell recognition mechanisms that determine the resistance or susceptibility of rubber trees to *Colletotrichum* are very scarce and require deep investigation.

Regarding the development of fungi at the temperatures studied, it was verified that this fungus can explore a wide range of temperatures; however, as observed by other authors [1,15,16], it has better growth in the plant at temperatures between 20 and 35 degrees. These temperatures are close to the average temperatures observed in most cities where rubber trees are grown in Brazil during most of the year [32]. This points to a difficulty in the development of disease prediction systems based on the temperature variable [33]. Therefore, the monitoring of rainfall can contribute to the management of this disease in the field, since longer periods of wetness directly influenced the percentage of spore germination and appressoria formation, as shown by Magalhães et al. [16].

## 4. Conclusions

The obtained results evidenced that *C. tamarillo* develops best in mild temperatures, but colonization and reproduction had a delay in resistant plants, which can lead to lower infection rates in the field, contributing thus to the management of this disease in rubber tree crops.

## Data Availability

The data presented in this study are available on request from the corresponding author. The data are not publicly available due to being part of a bigger project.
